# Virtual Screening for Potential Phytobioactives as Therapeutic Leads to Inhibit NQO1 for Selective Anticancer Therapy

**DOI:** 10.3390/molecules26226863

**Published:** 2021-11-14

**Authors:** Bhargav Shreevatsa, Chandan Dharmashekara, Vikas Halasumane Swamy, Meghana V. Gowda, Raghu Ram Achar, Vivek Hamse Kameshwar, Rajesh Kumar Thimmulappa, Asad Syed, Abdallah M. Elgorban, Salim S. Al-Rejaie, Joaquín Ortega-Castro, Juan Frau, Norma Flores-Holguín, Chandan Shivamallu, Shiva Prasad Kollur, Daniel Glossman-Mitnik

**Affiliations:** 1 Department of Biotechnology and Bioinformatics, School of Life Sciences, JSS Academy of Higher Education and Research, Mysuru 570015, India; bhargavshreevatsa@gmail.com (B.S.); chandand@jssuni.edu.in (C.D.); 2 Division of Biochemistry, School of Life Sciences, JSS Academy of Higher Education and Research, Mysuru 570015, India; hsvikas8@gmail.com (V.H.S.); meghanavgowda165@gmail.com (M.V.G.); 3 School of Natural Science, Adichunchanagiri University, B.G. Nagara, Nagamangala, Mandya 571448, India; vivekhamse@acu.ac.in; 4 Department of Biochemistry, JSS Medical College, JSS Academy of Higher Education and Research, Mysuru 570015, India; kumarrt@yahoo.com; 5 Department of Botany and Microbiology, College of Science, King Saud University, P.O. Box 2455, Riyadh 11451, Saudi Arabia; assyed@ksu.edu.sa (A.S.); aelgorban@ksu.edu.sa (A.M.E.); 6 Department of Pharmacology and Toxicology, College of Pharmacy, King Saud University, P.O. Box 55760, Riyadh 11451, Saudi Arabia; rejaie@ksu.edu.sa; 7 Departament de Química, Universitat de les Illes Balears, 07122 Palma de Malllorca, Spain; joaquin.castro@uib.es (J.O.-C.); juan.frau@uib.es (J.F.); 8 Laboratorio Virtual NANOCOSMOS, Departamento de Medio Ambiente y Energía, Centro de Investigación en Materiales Avanzados, Chihuahua 31136, Mexico; norma.flores@cimav.edu.mx; 9 Department of Sciences, Mysuru Campus, Amrita School of Arts and Sciences, Amrita Vishwa Vidyapeetham, Mysuru 570026, India

**Keywords:** NQO1, virtual screening, molecular docking, Nrf2, detoxification, phytobiactives, binding affinity, conceptual DFT

## Abstract

NAD(P)H:quinone acceptor oxidoreductase-1 (NQO1) is a ubiquitous flavin adenine dinucleotide-dependent flavoprotein that promotes obligatory two-electron reductions of quinones, quinonimines, nitroaromatics, and azo dyes. NQO1 is a multifunctional antioxidant enzyme whose expression and deletion are linked to reduced and increased oxidative stress susceptibilities. NQO1 acts as both a tumor suppressor and tumor promoter; thus, the inhibition of NQO1 results in less tumor burden. In addition, the high expression of NQO1 is associated with a shorter survival time of cancer patients. Inhibiting NQO1 also enables certain anticancer agents to evade the detoxification process. In this study, a series of phytobioactives were screened based on their chemical classes such as coumarins, flavonoids, and triterpenoids for their action on NQO1. The in silico evaluations were conducted using PyRx virtual screening tools, where the flavone compound, Orientin showed a better binding affinity score of −8.18 when compared with standard inhibitor Dicumarol with favorable ADME properties. An MD simulation study found that the Orientin binding to NQO1 away from the substrate-binding site induces a potential conformational change in the substrate-binding site, thereby inhibiting substrate accessibility towards the FAD-binding domain. Furthermore, with this computational approach we are offering a scope for validation of the new therapeutic components for their in vitro and in vivo efficacy against NQO1.

## 1. Introduction

**Figure 1:**
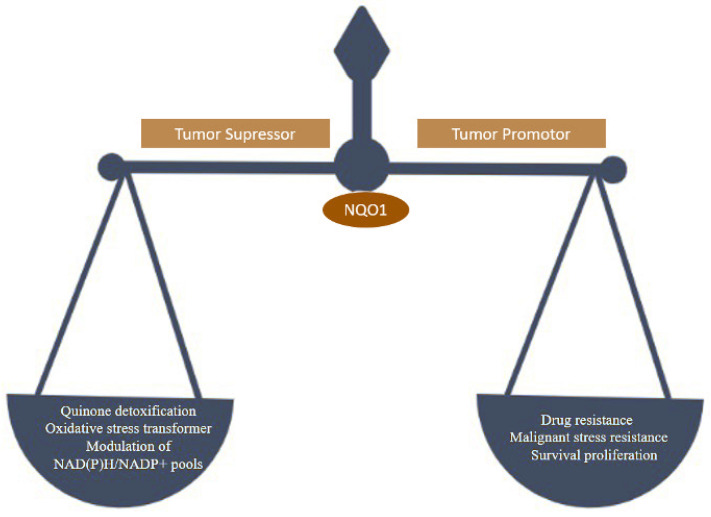
Tumor suppressor and tumor promoter behavior of NQO1 linked to reduced and increased oxidative stress susceptibilities.

NQO1 is one of the two main quinone reductases in the mammalian system. The paralog of cytosolic protein NQO1 is NQO2. NQO1 was isolated in the late 1950s, identified in rat liver, and named DT-diaphorase (DTD) by Lars Ernster. It is highly inducible in rat liver by azo dyes and polycyclic aromatic hydrocarbons regulated by the Keap1/Nrf2/ARE pathway and plays various roles in cellular stress response, including oxidative stress [1]. Under stress conditions, the NQO1 levels can increase rapidly, presumably as a cellular protective system [2]. NQO1 is a ubiquitous soluble enzyme found in almost all animal species. A wide range of inducers, including oxidants and phenolic compounds, are highly effective in inducing NQO1 [3]. Utilizing NAD(P)H as an electron donor catalyzes two-electron reduction of quinones to hydroquinone. NQO1 binds and stabilizes several short-lived proteins, including the tumor suppressors p53 and p73 and the enzyme ornithine decarboxylase (ODC) [4]. NQO1 is present in almost all normal human tissues at different levels of expression. In general, adipocytes, vascular endothelium, and epithelial cells express it at high levels. Human hepatocytes and miocardia have lower levels of NQO1 expression [5,6]. The NQO1 enzyme is a 273-amino-acid protein regulated by Nrf2 and the aryl hydrocarbon receptor and encoded by the NQO1 gene (AhR) [7]. Its function has been extensively researched. NQO1 has been studied extensively for its quinone reduction, significant antioxidant activity, reduction of ubiquinone and vitamin E derivatives to antioxidant forms, and superoxide scavenging [2]. Apart from its functions in disease models, NQO1 has also been linked to human cardiovascular diseases [8]. The multifunctionality of the NQO1 is represented in Figure 1.

NQO1 is a promising target for cancer detection, selective anticancer therapy, and provides multiple layers of protection to cells against carcinogenesis. NQO1 is involved in shielding cells from a range of harmful compounds, including quinones and other reactive oxygen species (ROS). Apart from this, it also plays a significant role in p53 stabilization [9]. This enzyme is bound to protect the cell’s internal molecules and prevent detrimental changes that disturb the cell’s homeostasis. NQO1 can provide several layers of protection to normal cells, allowing them to resist carcinogens [10].

**Figure 2:**
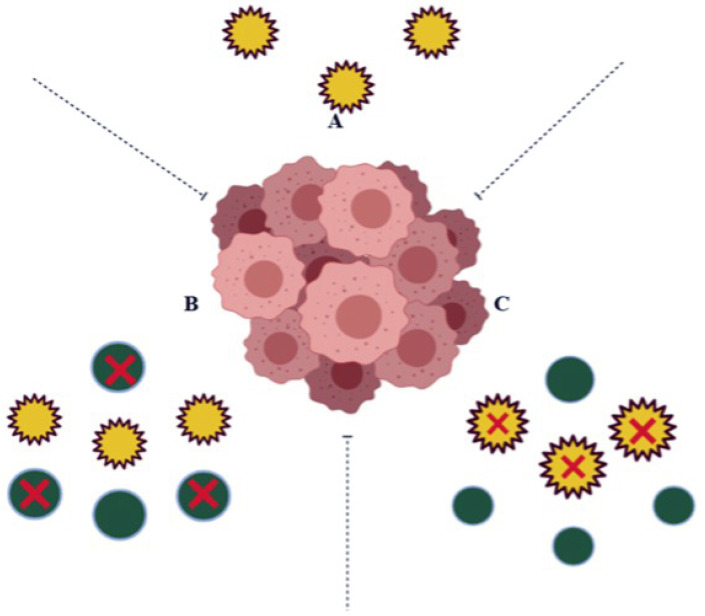
In some cases, (**A**) the expression levels of NQO1 (yellow circles) in a normal cell play a vital role in protecting the cells against harmful components; (**B**) in cancer cells, the over expression of NQO1 detoxifies Quinine molecules (green circles) resulting as Quinone resistant; (**C**) the inhibition of NQO1 in cancer cells avoids Quinone detoxification and shows susceptibility to anti-cancerous agents.

In cancer cells, it is over expressed, encouraging cell proliferation, malignant transformation, and drug resistance. Hence NQO1 acts as both a cancer suppressor and tumor promoter (Figure 2) [11].

**Figure 3:**
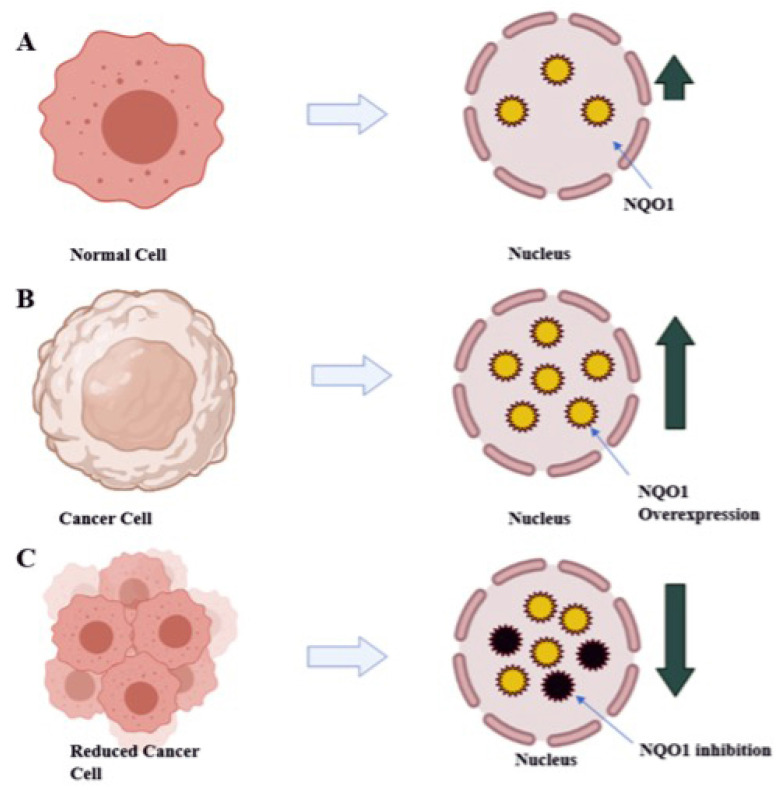
Diagrammatic representation of therapeutic approach with reduction of NQO1 levels in tumor cells results in decreased tumor burden: (**A**) normal liver expressing NQO1 (yellow circles) at normal level; (**B**) NQO1 (yellow circles) over expressed in tumor cells; (**C**) treating with anti-NQO1 drugs leads to the decreased tumor burden by decreased level of NQO1 (inhibited NQO1 black circles).

The high amount of NQO1 in cancer cells is thought to help them cope up with oxidative stress, just as they do in healthy cells. Studies of transcription factor Nrf2, which controls NQO1 expression, have partially supported this hypothesis. Gain-of-function mutations in Nrf2 or loss-of-function mutations in Keep over-activate Nrf2 in many cancers, including lung, pancreas, liver, gall bladder, and ovarian cancers [12]. Nrf2 gene hyperactivation helps malignant or transformed cells escape severe oxidative stress like over expression of NQO1. The mechanism by which NQO1 is over expressed via Nrf2 may vary between solid tumor types. For example, in gall bladder cancer, mutations in Keap1 cause the lowest binding with Nrf2 and drive the expression of NQO1. In contrast, in pancreatic cancer, K-ras mutations cause the lowest binding with Nrf2 and drive the expression of NQO1 [13]. Furthermore, Nrf2-induced over expression of NQO1 can play a significant role in chemotherapy failures, where it may be an adaptive response to oxidative stress and cytotoxicity and provide cancer cells with defense [14]. Therefore, as depicted in Figure 3, inhibiting NQO1 would enable selective anticancer therapy for chemotherapy failures caused due to detoxification catalyzed by NQO1 [14].

Ayurveda, the Indian system of medicine, is one of the earliest systems of medical practice in health management and has played a vital role in providing health care service to human civilization from its inception. Medicinal plants are still the essential source of modern medicine. Additionally, the literature reveals that Indian traditional plants have high potent phytobioactive compounds for developing new drugs [15,16]. The medical substances causing “a state of non-specifically increased resistance” of the organism (SNIR) were named “adaptogens”. A plant-origin substance increases non-specific resistance [17,18]. Development of phytobioactives that can move from the state of immune deficiency to normal condition would likely have a significant impact [19].

The proposed study was primarily focused on quinones, but the chemical classes like flavones, curcumin, and coumarins were previously reported as the potent inhibitors for NQO1. The flavonoids are identified as a potent inhibitor for NQO1 through competitive inhibition of NAD(P)H [20,21,22]. The quinones and related molecules like flavones, terpenoids, coumarins from tulsi, garlic, turmeric, punarnava, and other various medicinal plants which have been potential anti-cancerous effects were short-listed for studying their plausible interaction with NQO1. Furthermore, the study was extended towards the selective phytobioactives with high specificity for cancer biology, and they are efficiently targeted to tumor tissues enhancing NQO1 [23].

The inhibitors of NQO1 have shown promising outcomes in certain aspects where dicoumarol, a competitive inhibitor of NQO1, potentiates cisplatin-induced apoptosis in p53 wild-type urogenital cancer cell lines [24]. Meanwhile, a study has also revealed that β-lapachone from the bark of the lapacho tree affects the expression of NQO1 by mediating the inactivation of the Akt/mTOR pathway, resulting in significant anti-proliferation and anti-metastasis effects in breast cancer cell lines [25]. Based on this evidence, we hypothesize that inhibition of NQO1 will sensitize cancer cells to chemotherapeutic drugs. The proposed in silico work is geared towards screening, identification, and biological characterization of phytochemical inhibitors of NQO1 for enhancing the anticancer activity of chemotherapeutics. In the current study, we have identified the interactions of several Indian medicinal plants containing phytobioactives, including quinones with the different residues of NQO1, and the conformational changes imposed upon their binding by using the Molecular Docking technique. The potency of the phytobioactives was evaluated by MD simulation studies.

## 2. Materials and Methods

### 2.1. Collection of Phytobioactives

Medicinal plants rich in quinones, flavones, and terpenoids were noted and their phytoconstituents were shortlisted accordingly. A list of biologically phytobioactives is depicted in Table 1.

### 2.2. Preparation of Ligand

**Figure 4:**
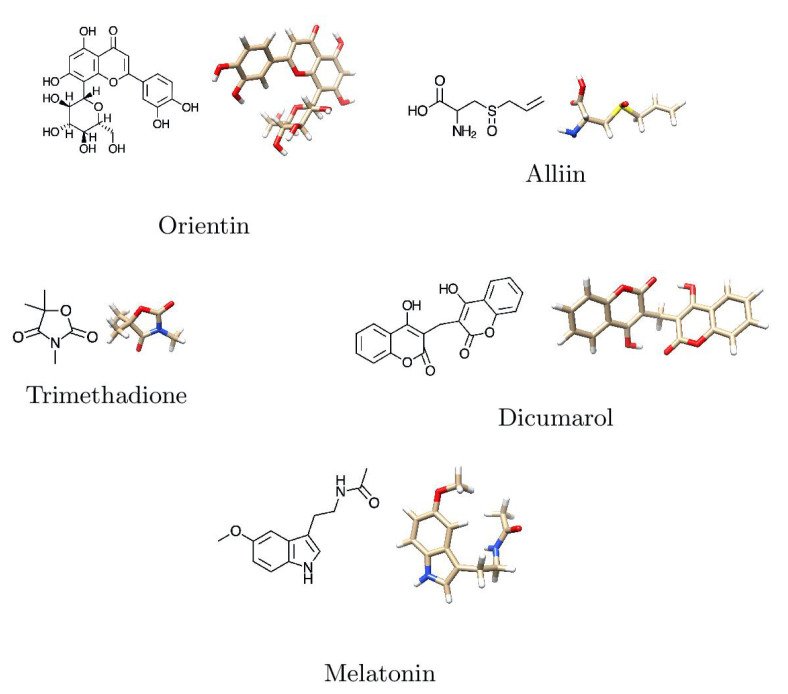
Two-dimensional and three-dimensional structures of major phytobioactives.

The selected ligands were downloaded from the NCBI, USA—PubChem chemistry database in three-dimensional form. The molecular docking software Argus Lab version 4.0.1 (http://www.arguslab.com/, (accessed on 11 August 2021)) was used to improve the geometric augmentation of the ligands. The two-dimensional and three-dimensional structures of prepared ligands are shown in Figure 4 and Table 2.

### 2.3. Protein Preparation

**Figure 5:**
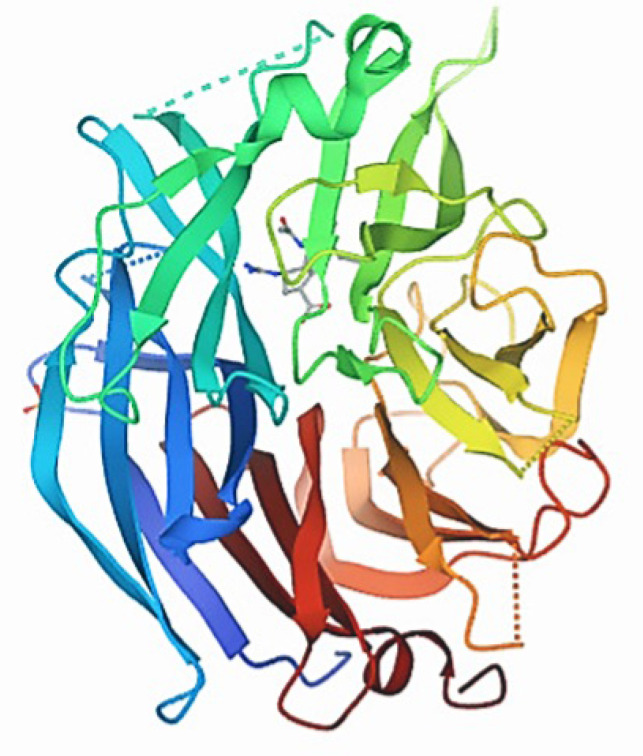
Three-dimensional structure of the protein NQO1 (PDB ID: 2F1O).

The three-dimensional Structure of the protein NQO1(PDB ID:2F1O) was retrieved from the Protein Data Bank (PDB). Furthermore, the non-standard amino acids bound to the downloaded NQO1 PDB file protein were removed or modified using Pymol software (Figure 5).

### 2.4. Prediction of the Binding Pockets

The binding pockets of NQO1(2F1O) protein-ligand binding were predicted using the in silico tool CASTp (http://sts.bioe.uic.edu/in, (accessed on 11 August 2021)).

### 2.5. Protein Refinement and Structure Validation

**Figure 6:**
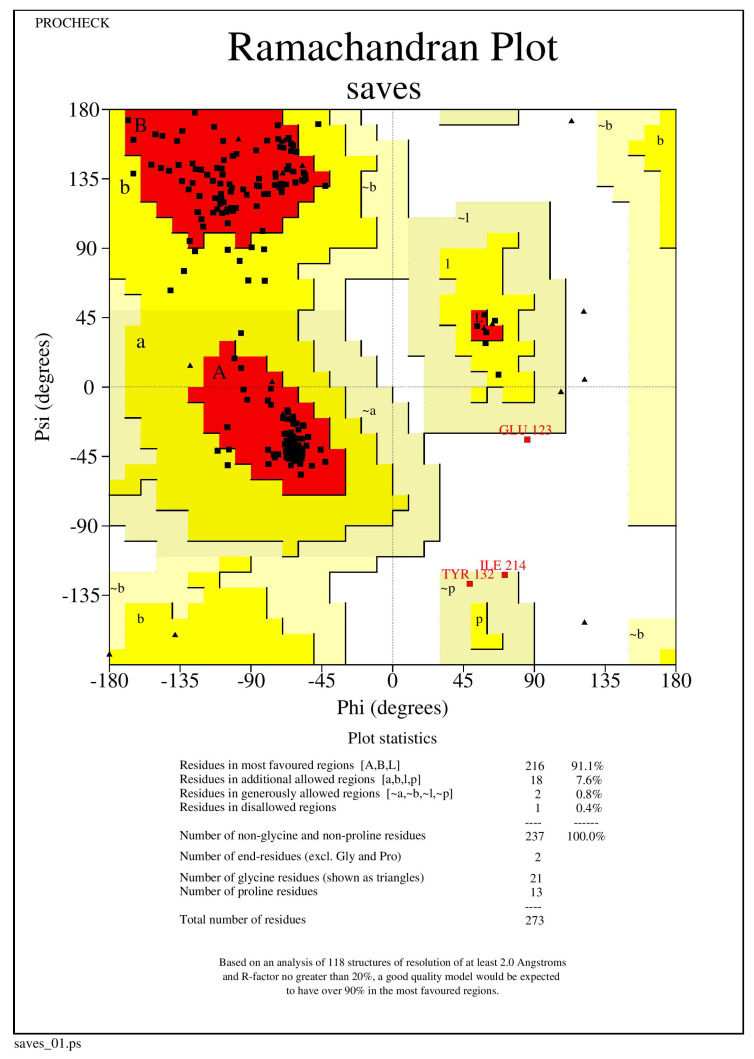
The Ramachandran plot generated from RAMPAGE. The Ramachandran plot representing energetically allowable regions for backbone dihedral angles ψ vs. ϕ amino acid residues in NQO1 (2F1O) protein structure.

The NQO1 (2F1O) protein structure was refined using the Mod Refiner server for protein refinement (https://zhanglab.ccmb.med.umich.edu/ModRefiner/, (accessed on 11 August 2021)). The Ramachandran plot was used to validate and evaluate the refined protein structures of NQO1, revealing that the energetically allowed protein structure of 2F1O regions for backbone dihedral angles toward amino acid residues were found. RAMPAGE was used to build the plots. The PROCHECK RAMPAGE results for the NQO1 revealed that its structure is stable, and the results are shown in Figure 6 and Table 3.

### 2.6. Molecular Docking Studies

The prix version 0.8 open access docking program was used for the better understanding of molecular interaction and the docking between the NQO1 protein and phytobioactives (ligands) were uploaded. The PDB format NQO1 protein was selected, the ligands were uploaded, and the grid box was marked to shield the active site residues, ready to be preferred binding residues to achieve the maximum orientation with the lowest binding affinity (Kcal/mol) values.

### 2.7. Molecular Docking Visualization

The Ligplot+2.2 and the BIOVIA Discovery Studio Visualizer were used to visualize the docked conformation of the ligand against the NQO1 protein. The interactions like hydrogen bonds, hydrophobic interaction, and bond length were visualized using this program, including the NQO1 protein and a variety of plant-derived compounds with some naturally occurring quinones. The 3D Visualization was done with the BIOVIA Discovery Studio Visualizer, while the 2D interaction was done with Ligplot+2.2 [26,27,28,29].

### 2.8. Molecular Dynamics

In the present study, a molecular dynamics simulation was carried out on a 64-bit Ubuntu 20.04 platform, in a Dell Precision 7820 equipped with Intel Xeon Gold (20 core), 512 GB RAM, and 24 GB Quadro RTX-6000 Nvidia GPU. MDSs for Orientin were carried out against NQO1 proteins. The water model was inserted in the docked protein-ligand complex in an orthorhombic periodic border of the box under solvated conditions using a system-builder such as TIP3P (transferable intermolecular potential with three points). To neutralize the system, Na^+^ ion (51.216 mM, with the charge of +31) and Cl^−^ (56.173 mM, with a charge of −34) for NQO1 was used. This step automatically neutralizes the charge of the system during the study of the protein dynamics when the ligand is competing at the binding site [30,31]. Furthermore, a molecular dynamics simulation was carried out under some periodic boundary conditions in the atom numbers, pressure, and temperature (NPT) ensemble, with the temperature set to 300 K and 1 atmospheric pressure, then relaxed using the Desmond program’s default relaxation methodology. The simulation job took 50 nanoseconds to complete. RMSD (Root-mean-square deviation), RMSF (Root-mean-square fluctuation) and the total energy of complexes were analyzed by using event analysis and simulations-interaction diagrams [32].

### 2.9. ADMET

**Figure 7:**
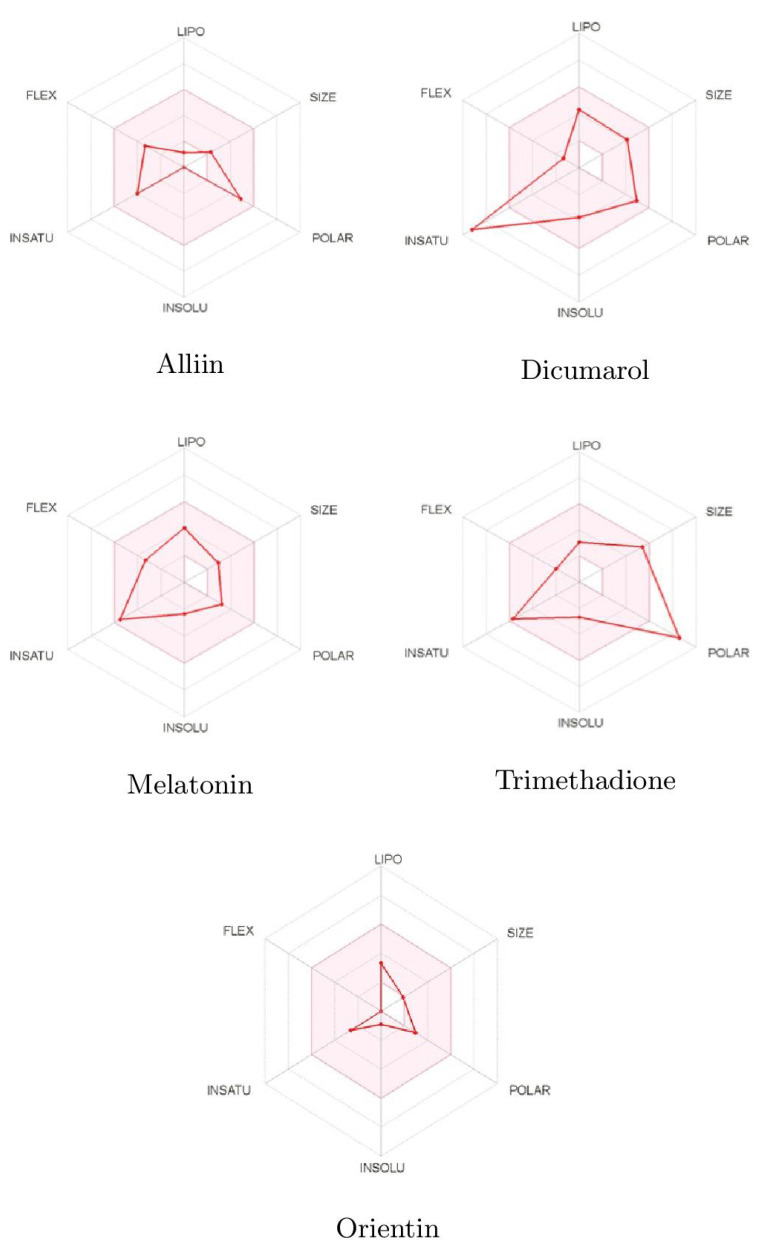
Bioavailabity radars of phytobioactives based on physicochemical indices with ideal values for oral bioavailability.

Pharmacokinetic and Toxicity (ADMET) profiles of identified phytobioactives from various medicinal plants were undertaken. It is generally established that poor ADMET (absorption, distribution, metabolism, excretion, and toxicity) qualities can degrade pharmacological activity. Furthermore, ADMET characteristics were examined using in silico methods to determine whether the screened phytobioactives would be good candidates for suitable medication. Unwanted pharmacokinetics and toxicity are practical causes of drug failure. This is expensive to discover at the clinical phase. The toxicity profile of all screened phytobioactives from various medicinal plants is based on AMES toxicity. Based on the bioavailability and drug-likeness of these three compounds, the results of more potent phytobioactives suitable for drug discovery are shown in Figure 7 and Table 4.

### 2.10. Conceptual DFT Studies

The molecular energy, electronic density, and orbital energies of a particular system, including the Highest Occupied Molecular Orbital (HOMO) and the Lowest Unoccupied Molecular Orbital (LUMO) were determined using the Kohn-Sham (KS) approach [33,34,35,36] considering the CDFT or conceptual density functional theory variant of DFT [37,38,39,40,41,42,43]. The conformers of the compounds studied in this work were determined using MarvinView 17.15 from ChemAxon [http://www.chemaxon.com], (accessed on 15 August 2021) by using the entire MMFF94 force field to perform molecular mechanics calculations [44,45,46,47,48]. The Density Functional Tight Binding (DFTBA) methodology [49] was considered for geometry preoptimization and frequency calculation. This step was required to ensure that there were no imaginary frequencies, which is a usual test for the optimized structures’ stability as a minimum within the energy surface. The estimation of the chemical reactivity descriptors of the studied ligands was accomplished using the MN12SX/Def2TZVP/H2O model chemistry [50,51,52] on the optimized at the same level molecular structures, as it has been shown that it fulfils the ’Koopmans in DFT’ (KID) protocol [53,54,55,56,57,58,59,60,61,62,63,64,65,66,67]. Gaussian 16 [49] and the SMD solvent model [68] were considered for the determinations. This model chemistry is based on the application of the MN12SX density function [50] in connection to the Def2TZVP basis set [51,52] being that the charge of the molecules is equal to zero and considering the corresponding negative and positive ions in the doublet spin state.

## 3. Results and Discussion

### 3.1. Protein Structure Validation

The NQO1 protein structure validation was examined using RAMPAGE. The initial validation was 92.4% of residues in a favorable region, and thus the protein could be subjected to further docking.

### 3.2. Protein-Ligand Interaction

**Figure 8:**
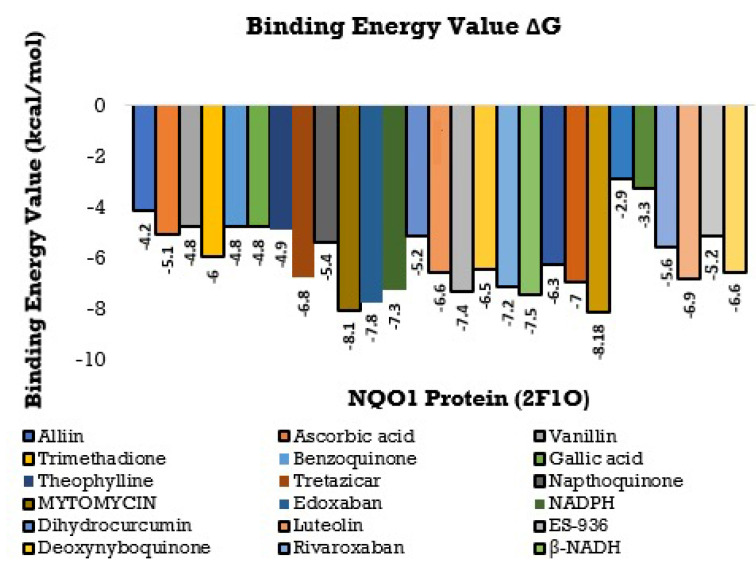
Histogram showing the Molecular Docking results between NQO1 (2F1O) against selective phytobioactives (the binding energy value δG is shown in minus kcal/mol).

**Figure 9:**
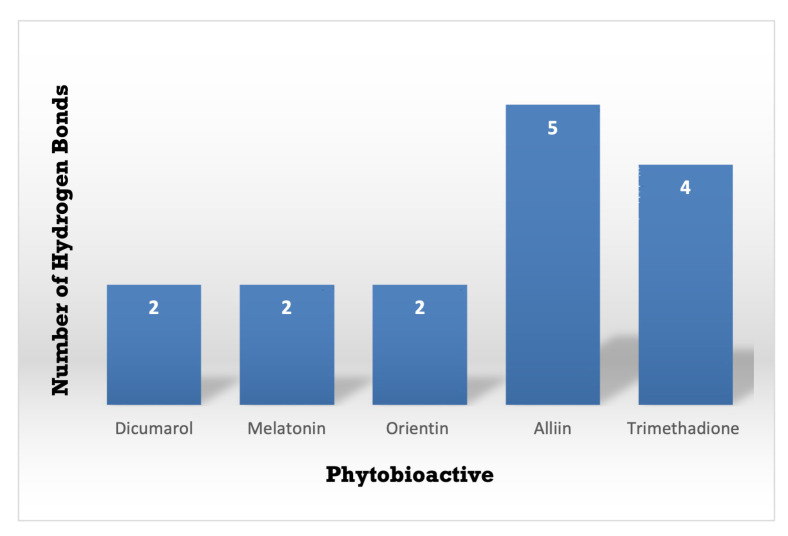
A Comparative study of the standard NQO1 inhibitor Dicumarol with the other potent phytobioactives. The X-Axis: Phytobioactives v/s Y-Axis: Number of hydrogen interactions towards protein.

The consequences of this study can be deciphered from Figure 8 and Figure 9 and, the best-docked posture of the multitude of sub-atomic docked compounds was thought about, and the most reduced relating restricting liking was marked. These docked molecules were visualized and analyzed utilizing Ligplot+ V 2.2 and BIOVIA Discovery studio Visualizer software, highlighting the nearby labeled binding residues [69]. The NQO1 protein (2F1O) is seen sharing the hydrogen bonds, which are lower than 2.75 long, with semi-surrounded hydrophobic associations with the chosen molecules.

### 3.3. Molecular Docking Interactions of 2F1O Protein with Selected Phytobioactives

**Figure 10:**
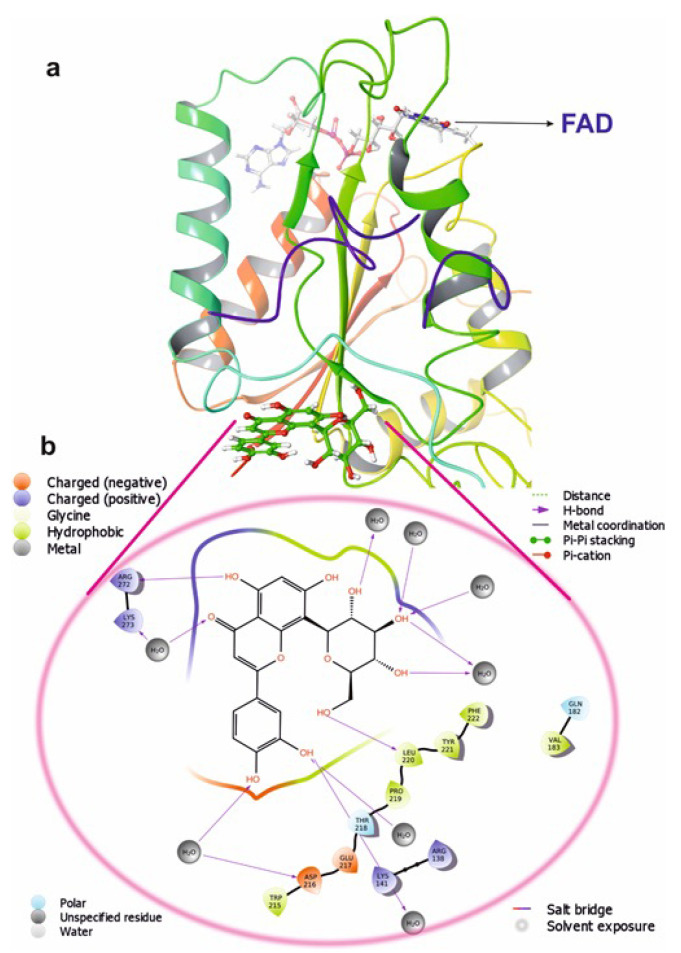
The putative binding pose of Orientin with NQO1: (**a**) Orientin represented in green can be seen deeply embedded into the active site. (**b**) The two-dimensional geometrical pose of Orientin interacting with surrounding amino acids at the putative binding cavity of NQO1.

Molecular docking is a commonly used approach in drug discovery for drug discovery with little or no adverse effects. It is a two-step process that begins with geometrical optimization of the ligand and target biomolecule. The conformations in the receptor-identified active region are then ranked according to their score, which varies from one software to the next depending on the algorithms used to develop them. Among the selected compounds, Orientin was found to be more potent against NQO1. All the listed compounds in Table 1 interacted with NQO1, but Orientin bound away from the coenzyme such as FAD binding to NQO1 (Figure 10), resulting in the non-competitive type of inhibition. Orientin interacts with NQO1 via hydrogen bond with Arg272, Lys141, and plenty of water molecules present at Orientin’s non-competitive bind site (Figure 10a,b). These results implicate that Orientin binding with NQO1 is very strong as compared to other compounds (see Table S2 of the Supplementary Materials).

**Figure 11:**
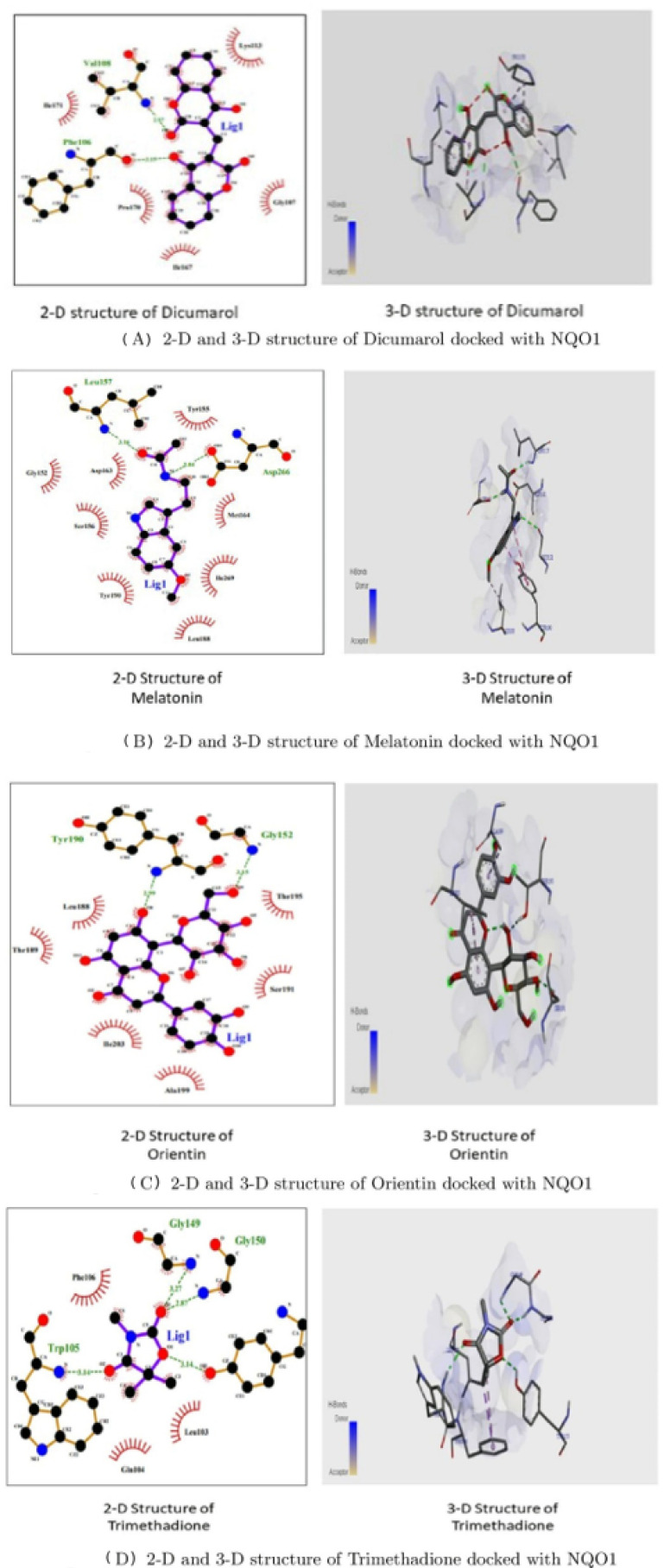
2-D and 3-D structures of several phytobioactives docked with NQO1.

Figure 11 shows that the two-Dimensional and three-Dimensional visualisation of the phytobioactives docked against the NQO1 (2F1O) protein exhibits an interaction, i.e., hydrogen bonding and other lower interactions like Van der Waals force, π−π stacked, π−π T-shaped, π-alkyl and carbon-hydrogen bond.

Using the Molecular Docking (in silico) technique, we analyzed the interaction between the NQO1 protein and a ligand molecule. Dicumarol is known as a potent (standard) inhibitory molecule for NQO1. The Dicumarol (Figure 11A) molecule (ligand) exhibits a binding affinity of −6.9 Kcal/mol and two hydrogen bonds with Phe 106 and Val 108 binding residues NQO1 protein, respectively. It also forms other interactions with the NQO1 protein. Similarly, melatonin (Figure 11B) exhibits a binding affinity of −5.6 Kcal/mol and forms two hydrogens bonding with Lue157 and Asp 266, respectively. Orientin (Figure 11C) shows the binding affinity of −6.6 Kcal/mol and forms two hydrogen bonds with the binding residues Gly52 and Tyr190. Trimethadione (Figure 11D) exhibits a binding affinity of −6 Kcal/mol and forms four hydrogen bonds with the binding residues Trp105, Trp105, Gly149, and Gly150. The binding to Gly150 is in accordance with previous results [27]. Also, in a recent study of the dual inhibitors NQO1 and GSTP1 (glutathione-S.transferase Pi1), MNPC was in alignment with our findings as far as some of the major amino acid residues are concerned [70].

### 3.4. Molecular Dynamics Simulations

**Figure 12:**
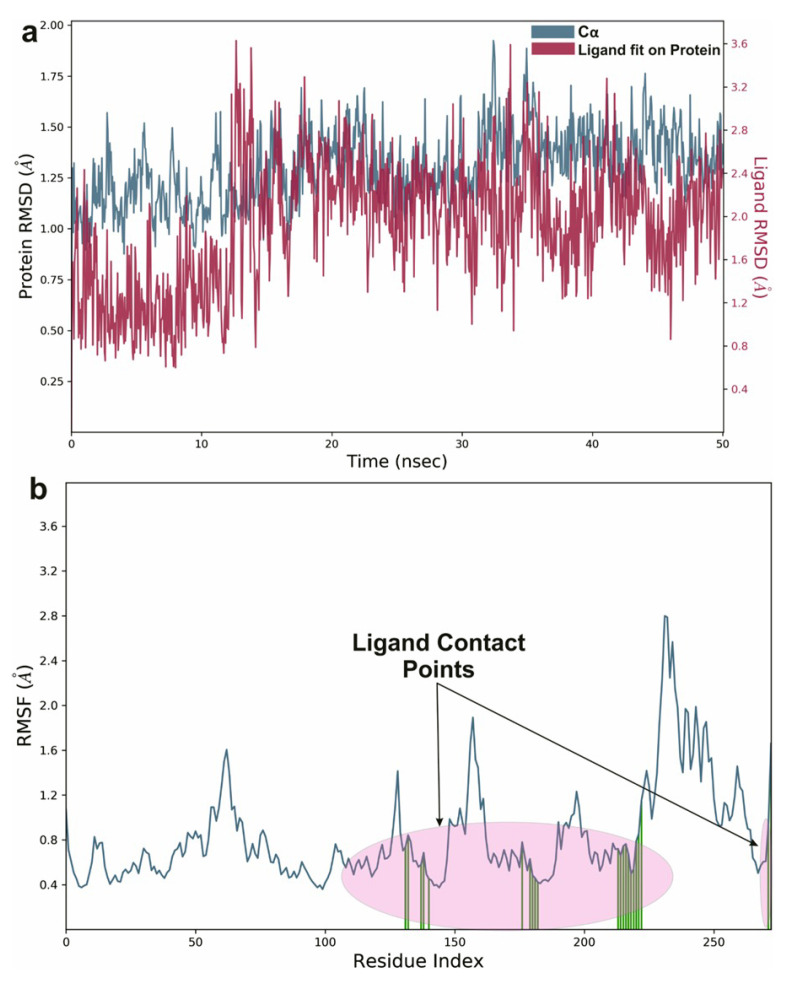
Graphical representation of (**a**) root-mean-square fluctuation for Orientin with active site residues of NQO1 after MDS and (**b**) ligand RMSD plot to show the contact point pf the ligand with the amino residue of the NQO1 represented as green color vertical green lines with several contact points.

**Figure 13:**
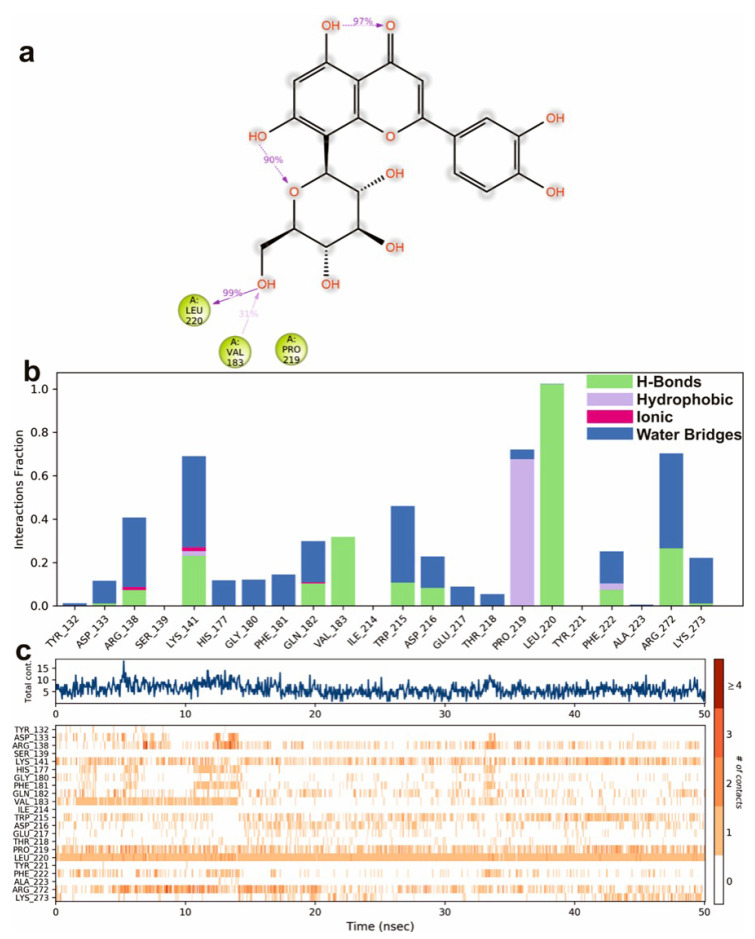
A scheme of detailed Orientin atom interactions with the protein residues. (**a**) Interactions that occur more than 30% of the simulation time in the selected trajectory. (**b**) Normalized stacked bar chart of compound Orientin interacting with NQO1 away from active site pocket through a hydrogen bond, hydrophobic and ionic interactions, and water bridges, and (**c**) The number of contact points with the amino acid residues are depicted in timeline representations coded with color intensity graphs throughout the simulation.

**Figure 14:**
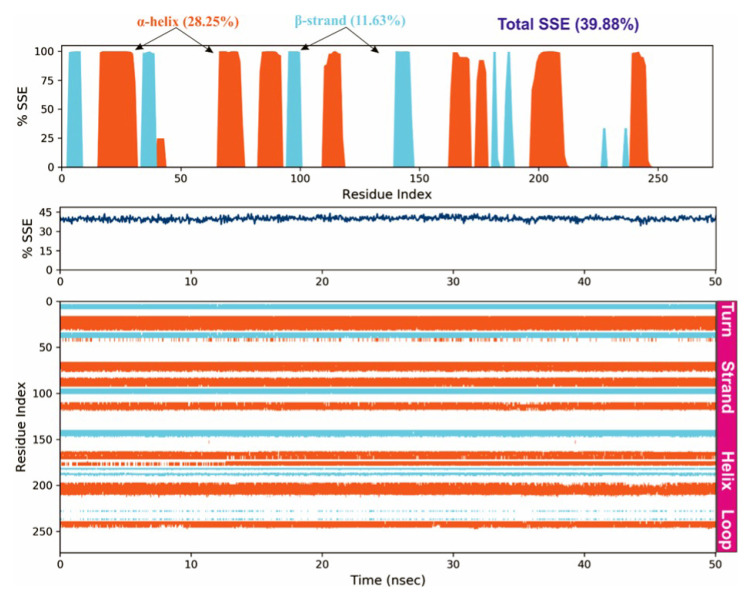
Protein secondary structure elements (SSE) like α-helices and β-strands are monitored throughout the simulation. The plot above reports SSE distribution by residue index throughout the protein structure.

To study the protein dynamics when the Orientin binds to NQO1, MDS studies were carried out to analyze the changes in protein during simulation for the docked protein-ligand complex. The Desmond equilibrates the system to achieve a stable conformation if the initial structure was energetically unstable. Hence in the present study, we performed MDS for docked complexes of Orientin and NQO1 (Figure 12). The protein RMSD overlaps with ligand RMSD (orientin) in the initial stage and goes up to 50 ns. It shows that it forms multiple interactions with the amino acid residues at the active site (Figure 12b). The type of interaction and number of the contact point is summarized in the pictorial diagram (Figure 13). The critical amino acids which aid in forming interactions are Leu220, Val183, and Pro219, and they play a prominent role at the non-competitive binding site. The two-dimensional binding geometry of Orientin with NQO1after MDSs shows that the Orientin interacts with the putative binding site of NQO1 with various interactions (Figure 13a–c). The protein secondary structure elements (SSE) for orientin binding to NQO1 induces the fluctuation in protein structure, which causes 28.25%, and 11.63% changes in α-helix and β-strands throughout the simulation up to 50ns (Figure 14). The graph below summarizes the SSE composition for each orbital frame during simulation, and the graph at the bottom monitors each residue and its SSE assignment over time (Figure 14). Thus, MDS studies suggest that Orientin binding to NQO1 away from the substrate-binding site induces a potential conformational change in the substrate-binding site, thereby inhibiting substrate accessibility towards the FAD binding domain.

### 3.5. Conceptual DFT Studies

The calculated global reactivity descriptors [37,38,39,40,41,42,43], which were estimated following the methodology presented in the Section 2.10 together with the in-house developed CDFT software tool, are displayed in Table 5.

Because global hardness is a direct measure of electron density deformation and chemical reactivity, which is related to the HOMO-LUMO gap, it can be seen that Orientin will be the most reactive ligand, with Dicumarol and Melatonin being very similar in reactivity, and Trimethadione will be the least reactive of all the ligands considered. The electrodonating power EDP is more important than its electroaccepting EAP counterpart for all the ligands, which can be explained in terms of their molecular structures. However, when the values of EDP and EAP for each molecule are compared, it can be deduced that the Orientin and Dicumarol ligands will have considerably different reactivity than the other ligands. The electrophilicity ω index encompasses the equilibrium between an electrophile’s tendency to acquire extra electron density and a molecule’s resistance to exchanging electron density with the environment [71]. According to an electrophilicity ω scale for classifying organic molecules as strong, moderate, or marginal electrophiles (>1.5 eV for the first case, between 0.8 and 1.5 eV for the second case, and 0.8 eV for the last case) [72,73,74] and a review of Table 5, all ligands can be classified as strong electrophiles with the exception of Trimethadione.

**Figure 15:**
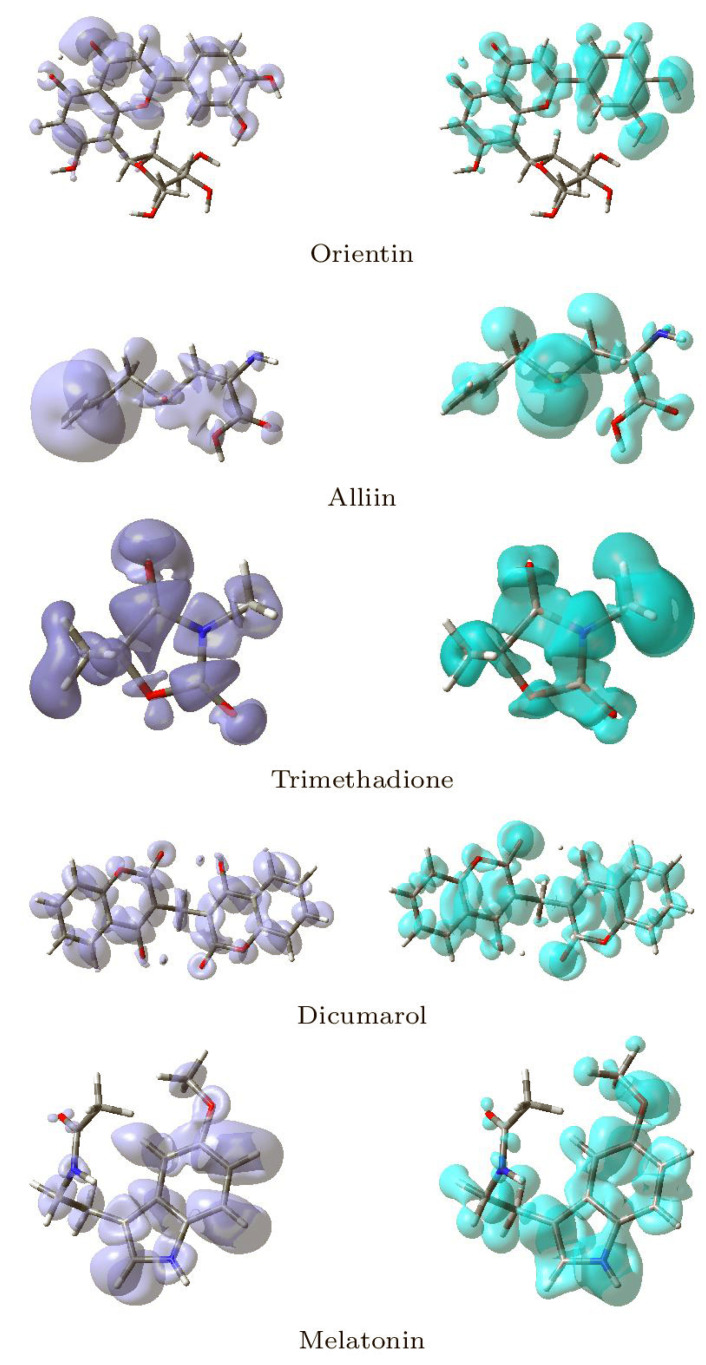
Graphical Representations of the Dual Descriptor DD of the Five Studied Ligands. (**Left**): DD > 0, (**Right**): DD < 0.

Local reactivity descriptors have been developed in addition to global reactivity descriptors to gain a sense of the changes in chemical reactivity across the atoms inside the molecule. The Fukui functions [37,38,39] and the dual descriptor [41,75,76,77,78,79] are the most commonly used local reactivity descriptors. The NFF and the EFF are associated with the sites within a molecular system which are prone to nucleophilic or electrophilic attacks, respectively. Although the NFF and EFF have proven to be beneficial in identifying reactive sites, the dual descriptor DD is thought to describe the nucleophilic and electrophilic sites in a molecule clearly [79]. Graphical representations of the DD for the five studied ligands is displayed in Figure 15 showing the zones where DD > 0 and DD < 0.

Although there is some overlap between the different sections inside the ligands, these graphical representations allow for a clear distinction between the locations within the molecules where the Dual Descriptor will be bigger or smaller than zero, signaling chemical reactivity differences.

## 4. Conclusions

In summary, the phytoactive compounds such as Orientin, Trimethadione, Alliin, and some other potent plant-derived molecules from a range of chemical classes has shown better interaction towards the NQO1 protein when compared to the standard NQO1 inhibitor Dicumarol. Understanding the complex mechanism and functioning of NQO1 in cancer studies reveals that this protein exhibits as both tumor suppressor and tumor promoter under different tools. The molecular docking and molecular dynamics studies revealed that the phytobioactive, Orientin, exhibited higher interaction with NQO1. The Orientin is a flavone molecule with an anticancer property that also includes anti-inflammation, neuroprotective, radiation protective, vasodilation, cardioprotective, antiviral, antibacterial, antidepressant-like antiadipogenesis, antinociceptive, and various antioxidant properties [80]. The results obtained from the current study conclude that the critical amino acids which aid in forming interactions are Leu220, Val183, and Pro219, which play a prominent role at the non-competitive binding site. The studies on flavonoids have implicated their potency as an antitumor agent. From this, we can hypothesize that using these phytobioactives can affect the expression of NQO1 in cancer cells. The biochemical properties and the mechanism of these phytochemical constituents can be studied and analyzed by in vitro and in vivo studies. In conclusion, the Indian Ayurvedic plants consist of various types of quinone molecules, which have a potency towards NQO1 inhibition. Furthermore, it would be interesting in the future to analyze the biological data through pharmacophore modeling and 3D QSAR studies to elucidate and substantiate the mechanism of action in comparison with the results of molecular docking and molecular dynamic studies considering the standard inhibitor, Dicumarol.

## Figures and Tables

**Table 1:** Screening of phytobioactives against NQO1 protein.

SI	CID	Compound Name	SI	CID	Compound Name	SI	CID	Compound Name
1	637563	Anethol	21	460	Guaiacol	41	442882	Justicidin B
2	160512	Ar-Turmerone	22	6549	Linalool	42	637520	Methyl Cinnamate
3	9793905	Sallylcysteine	23	8815	Estragol	43	5280343	Quercetin
4	11128	Linamarin	24	10281	Thymoquinone	44	5280346	Ubiquinone
5	20657680	S-allylmercaptocystein	25	11617	Dyallyl Sulfide	45	5280445	Luteolin
6	10494	Oleanolic acid	26	29746	Geosmin	46	5280489	β-Carotene
7	64945	Ursolic acid	27	6654	α-Pinene	47	5281672	Myricetin
8	5315615	Rosemarinic acid	28	7794	Citronellol	48	5281675	Orientin
9	370	Gallic acid	29	65036	Allicin	49	5315472	Bisdemethoxycurcumin
10	10364	Carvacrol	30	70308	2,2-Dimethylpropiophenone	50	5386591	Ajoene
11	5281515	β-Caryophyllene	31	87014	Anthraquinone	51	7794	Citronellol
12	2537	Camphor	32	87310	Alliin	52	1042933	Dihydrocurcumin
13	2519	Caffeine	33	92139	α-Curcumin	53	1125088	β NADH
14	2758	Eucalyptol	34	323	Coumarin	54	12302243	α-Calacorene
15	3314	Eugenol	35	442793	Gingerols	55	17753965	Benzopyrone
16	3885	β-Lapachone	36	160512	Ar-Turmerone	56	54670067	L-Ascorbic acid
17	5281166	Jasmonic acid	37	167812	Curcumenol	57	643779	Neral
18	5429	Theobromine	38	196216	β-Turmerone	58	54736423	Dimethyl-3-naphtalene
19	5746	Mitomycin	39	442402	Thujopsene	59	31211	Zingerone
20	6616	Camphene	40	86895	Cuparene	60	117587706	Dihydronaphtalene

**Table 2:** Major plant derived phytobioactive constituents with plant source, scientific name and molecular formula.

SI No	Name of	Taxonomical	Chemical	Name of the Phytobioactive	Molecular Weight	Chemical
	the Plant	Name	Formula	Components	(g/mol)	Class
A	Holy Tulsi	Ocinum tenuiflorum	C_21_H_20_O_11_	Orientin	448.40	Flavone
B	Garlic	Allium sativum	C_6_H_10_OS_2_	Alliin	177.22	Tioester
C	Punarnava	Boerhavia diffusa	C_6_H_9_NO_3_	Trimethadione	143.14	Oxazolidinedione

**Table 3:** The values found from RAMPAGE represents the number of residues in favored region, allowed region and outliner regions through Ramachandran plot evaluation centre and the outliner regions through the RAMPAGE evaluation center.

SI No	Protein Structure	Number of Residues	Number of Residues	Number of Residues in
	of NQO1	in Favored Regions (%)	in Allowed Regions (%)	Disallowed Regions (%)
1	2F1O	92.4	6.8	0.0

**Table 4:** ADMET identified properties of top three phytobioactives together with Dicumarol and Melatonin.

ADMET Entry	Alliin	Dicumarol	Melatonin	Trimethadione	Orientin
	Drug Likeness				
Lipinski	Yes	Yes	Yes	Yes	No
Bioavailability Score	0.55	0.55	0.55	0.55	0.55
	Solubility				
Water Solubility	Yes	Yes	Yes	Yes	No
	Absorption				
Intestinal Absorption	High	High	High	High	Low
Skin Permeability	−9.14	6.88	6.59	−6.96	−9.14
P-glycoprotein Substrate	No	No	No	No	No
	Distribution				
BBB Permeability	No	No	Yes	No	No
CYP1SA2 Inhibitor	No	Yes	Yes	No	No
CYP12C19 Inhibitor	No	No	No	No	No
CYP2C9 Inhibitor	No	No	No	No	No
CYP2D6 Inhibitor	No	No	No	No	No
CYP3A4 Inhibitor	No	No	No	No	No
	Toxicity				
AMES Toxicity	No	No	No	No	No

**Table 5:** Global Reactivity Descriptors of the Five Studied Ligands (all in eV, excepting S, in eV${}^{-1}$).

Molecule	χ	η	ω	S	N	$\omega^{-}$	$\omega^{+}$	$\Delta \omega^{\pm }$
Orientin	4.15	3.73	2.31	0.27	2.78	6.93	2.78	9.71
Alliin	3.44	5.96	0.99	0.17	2.37	4.09	0.64	4.73
Trimethadione	4.43	7.16	1.37	0.14	0.78	5.41	0.98	6.39
Dicumarol	4.32	4.26	2.19	0.23	2.34	6.81	2.49	9.29
Melatonin	3.23	4.71	1.11	0.21	3.21	4.12	0.89	5.01

## Data Availability

All the data originated from this research is available from the authors under request.

## Supplementary Materials

The following are available online, Table S1: List of some synthetic and semi-synthetic compounds screened against NQO1; Table S2. Binding affinity and hydrogen bonding interaction of molecular docking analysis of phytobioactives against NQO1 (2F1O) Protein.
