# Supplementary material for: Virtual Screening for Potential Phytobioactives as Therapeutic Leads to Inhibit NQO1 for Selective Anticancer Therapy
Source: Molecules. 2021 Nov 14;26(22):6863. doi: 10.3390/molecules26226863 (PMC8622762; doi:10.3390/molecules26226863)
Supplement: Supplementary file 1 [file molecules-26-06863-s001.zip › molecules-1426699-supplementary.pdf]

Supplementary Data

Table S1. List of some synthetic and semi-synthetic compounds screened against NQO1.

1	2244	Aspirin
2	89105	Tretazicar
3	216210	Dabigatran
4	54678486	Warfarin
5	1183	Vanillin
6	98527	Dimethoxy-4-Bromophenetholamine
7	154482	Hexabromo-1,1 Biphenyl
8	332529	9-(3,4-Dimethoxyphenyl)-5-hydroxy-6H-[2]benzofuro[5,6-f][1,3]Benzodioxol-8-One
9	442664	Vicenin
10	442882	Justicidin
11	3633866	Naphthalen-2-Ylsulfonyl
12	4470790	ES-936
13	10397115	2-(Aziridin-1-yl)-5-methyl-1,4-benzoquinone
14	101916320	4-0-Demethyl Suchilactone

Table S2. Binding affinity and hydrogen bonding interaction of molecular docking analysis of phytobioactives against NQO1 (2F1O) Protein.

Sl No	Compound CID	Ligand	Binding Affinity Kcal/mol	Hydrogen bonding forming Amino Acid residues
**1**	87310	Alliin	-4.2	Lue 103, Tyr 155(2), Gly 150, Tpr 105
**2**	54670067	Ascorbic acid	-5.1	Phe 106, Lys A 113.
**3**	1183	Vanillin	-4.8	Ala 223.
**4**	5576	Trimethadi	-6	Trp 105 (2), Gly149,

		one		Gly 150.
**5**	11958	Benzoquinone	-4.8	Tyr 155, Trp 105.
**6**	370	Gallic acid	-4.8	Val 108.
**7**	2153	Theophylline	-4.9	Lue 103, Tyr 102.
**8**	89105	Tretazicar	-6.8	Lue 103, Gly 150.
**9**	16871	Napthoquinone	-5.4	Trp 105, His161, Tyr 155.
**10**	5746	MYTOMYCIN	-8.1	Trp 105, Gly 149.
**11**	10280735	Edoxaban	-7.8	His 11, His 194.
**12**	5884	NADPH	-7.3	Gln 187 (2), Pro 186, Lue 188 (2), Tyr 190, Gly 206, Arg 210, Lys 270.
**13**	10429233	Dihydrocurcumin	-5.2	Trp 105, Tyr 155.
**14**	5280445	Luteolin	-6.6	Pro 68, Gly 122.
**15**	4470790	ES-936	-7.4	Tyr 67.
**16**	295934	Deoxynybo quinone	-6.5	Gly 149, Tyr 105, Tyr 155, His 169.
**17**	9875401	Rivaroxaban	-7.2	Lys 270, Tyr 190
**18**	11250888	β-NADH	-7.5	Gly 149, Tyr 155.
**19**	117587706	Dihydronapthalene	-6.3	Tyr 126.
**20**	101916320	Demethyl Suchilaceone	-7	Ile 50, Lys 270.

**21**	5281675	ORIENTIN	-6.6	Tyr190, Gly150.
**22**	11617	DIALLYL SULFIDE	-2.9	Phe 106.
**23**	5386591	Ajoene	-3.3	Ile 164
**24**	896	Melatonin	-5.6	Leu 157, Asp 266.
**25**	54676038	Dicumarol	-6.9	Val 108, Phe 106.
**26**	4055	Menadione	-5.2	Lys 113.
**27**	5281672	Myricetin	-6.6	Phe 178, Pro 68.
